# Response from the Canadian Children Inflammatory Bowel Disease Network to the US Food and Drug Administration Draft Guidance for Industry on pediatric inflammatory bowel disease: developing drugs for treatment

**DOI:** 10.1093/jcag/gwae034

**Published:** 2024-10-12

**Authors:** Eytan Wine, Jennifer deBruyn, Eileen Crowley, Anne M Griffiths, the Canadian Children IBD Network, Jocelyn Jeong, Kevan Jacobson, Sally Lawrence, Hannah Piper, Matthew Smyth, Carolina Tropini, Bruce Vallance, Jennifer deBruyn, Eytan Wine, Hien Huynh, Matthew Carroll, Daniela Migliarese Isaac, Heather Armstrong, Wael El-Matary, Zubin Grover, Eileen Crowley, Kevin Bax, Herbert Brill, Jenna Dowhaniuk, Katherine Prowse, Mary Sherlock, Mary Zachos, Lara Hart, Cynthia Popalis, Anne M Griffiths, Aleixo Muise, Amanda Ricciuto, Eric Benchimol, Nicola Jones, Nicholas Carman, Peter Church, Thomas Walters, Daniel Mulder, David Mack, Joanna Stanisz, Alain Stintzi, Nikhil Pai, Colette Deslandres, Jessica Breton, Kelly Grzywacz, Prévost Jantchou, Laurence Chapuy, Rilla Schneider, Anthony Otley, David Burnett, Jeff Critch, Sarah Dinn

**Affiliations:** Canadian Children Inflammatory Bowel Disease Network, Canada; Division of Pediatric Gastroenterology, Department of Pediatrics, University of Alberta, Edmonton, AB T6G1C9,Canada; Canadian Children Inflammatory Bowel Disease Network, Canada; Division of Pediatric Gastroenterology, Department of Pediatrics, University of Calgary, Calgary, AB T2N1N4, Canada; Canadian Children Inflammatory Bowel Disease Network, Canada; Division of Pediatric Gastroenterology, Department of Pediatrics, University of Western Ontario, London, ON N6A5W9,Canada; Canadian Children Inflammatory Bowel Disease Network, Canada; Division of Pediatric Gastroenterology, Department of Pediatrics, University of Toronto and Hospital for Sick Children, Toronto, ON M5G1X8, Canada

Representing the majority of pediatric gastroenterologists treating children with inflammatory bowel diseases (IBD) in Canada, we would like to share with the readers of the Journal of the Canadian Association of Gastroenterology (JCAG) the content of a letter we submitted to the USA Food and Drug Administration (FDA). This letter (with minor modifications to provide context) was a response to a document (https://www.fda.gov/regulatory-information/search-fda-guidance-documents/pediatric-inflammatory-bowel-disease-developing-drugs-treatment#:~:text=The%20draft%20guidance%20provides%20FDA’s,efficacy%20considerations%2C%20and%20safety%20assessments) that ‘*provides FDA’s recommendations about the necessary attributes of clinical studies for drugs being developed for the treatment of pediatric ulcerative colitis or pediatric Crohn’s disease, including study population, study design, efficacy considerations, and safety assessments*’. Given the impact of trial design on care for Canadian children with IBD, we wanted CAG members and JCAG readers to be aware of this topic and hope to promote advocacy for our patients and public discussion.

“We read with great interest and anticipation the draft guidance document on developing drugs for the treatment of Pediatric IBD (PIBD) and were happy to learn that the FDA has invited feedback on this process. Although we are all practicing PIBD experts in Canada, and not in the United States, we felt that it would be imperative that we include our voice in this process, as decisions made by the FDA have a major impact on the design of global clinical trials we are invited to participate in, and eventually on therapies that are made available to our patients. We hope that this consultation process will result in a better process for drug approval for PIBD patients.

The Canadian Children IBD Network (CIDsCaNN) was established in 2013 (funded through grants from the Canadian Institutes for Health Research; CIHR, and the CHILD foundation) and now includes all 13 academic referral centres in Canada, and 70 members (realistically, all PIBD experts in Canada). CIDsCaNN (cidscann.ca) is involved in clinical and translational research, as well as education and advocacy, and has evolved as the leading platform in Canada for clinical care standards for PIBD patients. Through our executive and steering committees, we have discussed these issues and included our consensus response to the FDA guidance document.

*Rationale*: we have seen tremendous improvements in the management of PIBD over the last decades, but major challenges remain. While most IBD is diagnosed in adulthood, the fastest-growing incidence is in children (especially young children) and the extent and severity of the disease are higher in children than adults with IBD. As a result, this population with a clear need for new therapies, is unfortunately deprived of many of these, directly due to the challenges in drug approval. The rationale for a special process for drug approval for children is well understood, but the reality is that the 10+ years gap in approval has created a very challenging situation for patients. As a result, many children and teens with PIBD are either not receiving treatments that would be effective for them, or if they are, these are given off-label (a situation we try to avoid). Therefore, there is an urgent need to radically change the process of extrapolation of findings from adult studies, together with prioritizing and focusing pediatric drug trials in a different direction. Canadian centres have been central contributors to PIBD RCTs, but these are becoming more difficult to recruit and/or unethical for our patients.

**Major points of concern**:

Like other international PIBD colleagues, who have submitted responses to the FDA, our major concerns with the draft guidance document are as follows:

1. *Extrapolation of efficacy*: This is, in our eyes, the key issue to be addressed. While the FDA draft document does acknowledge the common pathophysiology between IBD in children and adults and provides good rationale for extrapolation, it does not go far enough in implementing this into action, despite similar, or even superior efficacy of approved biologics in pediatric studies. We call for *full extrapolation* of findings from adult studies. Essentially, we recognize the importance of conducting drug trials in children; however, these should not be designed to replicate findings in adults, but rather to focus on dosing (pharmacokinetics and pharmacodynamics) and differences in young children. We agree with the principles for extrapolation, as they are very well expressed in a letter from the Porto IBD Group of ESPGHAN (European Society for Pediatric Gastroenterology, Hepatology and Nutrition; paper under review).
2. *Questioning the frequency of repeated colonoscopic procedures*: Endoscopic examination is still considered the gold standard for assessment of disease status in IBD. There are, however, well-studied and validated non-invasive tools, including the MINI index, fecal calprotectin, and bowel ultrasound that should be used as surrogates at certain time points during a pediatric clinical trial. The use of surrogates early post-induction would allow restriction of colonoscopy to baseline and end of the study.
3. *Dosing in pediatric clinical trials*: Previous RCTs, and the current draft guidance document, support the comparison of 2 doses of an effective drug studied in adults. While we see benefits to this approach replacing the option of randomization with a placebo, we do not support the use of a low dose, not been shown to be effective in adults (in fact, multiple studies in children have shown that higher doses are required, especially for younger/smaller children).

We do hope that the FDA will address these concerns with full attention, recognizing the voices of serious discomfort with many aspects of the draft document and the internationally united message from leaders in clinical care and research for children with IBD. There is ongoing concern in the international pediatric IBD community that previous discussions with regulators have not resolved the challenging reality of more than a decade passing between drug approval in adults and children. As those who are eager to advance approval of new effective medications for our patients (who are in clear need of these), and those who work to recruit children to studies designed to allow drug approval, we would like to voice the difficult situation we are dealing with a slow process of approval of drugs for children and inability to recruit to recent studies. We are grateful for the opportunity to share our thoughts and would be delighted to continue this discussion, hoping to have a true impact on the process.”

This letter was drafted by the executive committee of CIDsCaNN (authors of this paper), with advice from other key members, and unanimously endorsed by the Canadian Children IBD Network membership.

## Supplementary material

Supplementary material is available at *Journal of the Canadian Association of Gastroenterology* online.

gwae034_suppl_Supplementary_Material

## Data Availability

There are no data associated with this article.

## Notes

Canadian Children IBD Network

Members of the Canadian Children IBD Network signing the letter include:

Jocelyn Jeong, Kevan Jacobson, Sally Lawrence, Hannah Piper, Matthew Smyth, Carolina Tropini, Bruce Vallance (University of British Columbia and BC Children's Hospital, Vancouver, British Columbia, Canada); Jennifer deBruyn (University of Calgary, Calgary, Alberta, Canada); Eytan Wine, Hien Huynh, Matthew Carroll, Daniela Migliarese Isaac (University of Alberta, Edmonton, Alberta, Canada); Heather Armstrong, Wael El-Matary (University of Manitoba, Winnipeg, Manitoba, Canada); Zubin Grover (Perth Children’s Hospital, Nedlands, Australia); Eileen Crowley, Kevin Bax (Children’s Hospital London Health Sciences Centre, London, Ontario, Canada); Herbert Brill, Jenna Dowhaniuk, Katherine Prowse, Mary Sherlock, Mary Zachos (McMaster Children's Hospital, Hamilton, Ontario, Canada); Lara Hart (Humber River Hospital, Toronto, Ontario, Canada); Cynthia Popalis (Boomerang Health, Toronto, Ontario, Canada); Anne M. Griffiths, Aleixo Muise, Amanda Ricciuto, Eric Benchimol, Nicola Jones, Nicholas Carman, Peter Church, Thomas Walters (The Hospital for Sick Children, Toronto, Ontario, Canada); Daniel Mulder (Queen’s University and Kingston Health Sciences Centre, Kingston, Ontario, Canada); David Mack, Joanna Stanisz, Alain Stintzi (University of Ottawa, Ottawa, Ontario, Canada); Nikhil Pai (Children’s Hospital of Philadelphia, Philadelphia, PA, United States); Colette Deslandres, Jessica Breton, Kelly Grzywacz, Prévost Jantchou, Laurence Chapuy (CHU Sainte-Justine, Montreal, Quebec, Canada); Rilla Schneider (McGill University, Montreal, Quebec, Canada); Anthony Otley, David Burnett (IWK Health Center, Halifax, Nova Scotia, Canada); Jeff Critch, Sarah Dinn (Memorial University, St. John’s, Newfoundland, Canada).

