# Supplementary material for: Response from the Canadian Children Inflammatory Bowel Disease Network to the US Food and Drug Administration Draft Guidance for Industry on pediatric inflammatory bowel disease: developing drugs for treatment
Source: J Can Assoc Gastroenterol. 2024 Oct 12;7(6):397–8. doi: 10.1093/jcag/gwae034 (PMC11637986; doi:10.1093/jcag/gwae034)
Supplement: gwae034_suppl_Supplementary_Material [file gwae034_suppl_supplementary_material.pdf]

ICMJE DISCLOSURE FORM

**Date:**	9/4/2024
**Your Name:**	Anne M Griffiths
**Manuscript Title:**	Response from the Canadian Children IBD Network to the US FDA Draft Guidance for Industry on Pediatric Inflammatory Bowel Disease: Developing Drugs for Treatment
**Manuscript Number (if known):**	Click or tap here to enter text.

In the interest of transparency, we ask you to disclose all relationships/activities/interests listed below that are related to the content of your manuscript. "Related" means any relation with for-profit or not-for-profit third parties whose interests may be affected by the content of the manuscript. Disclosure represents a commitment to transparency and does not necessarily indicate a bias. If you are in doubt about whether to list a relationship/activity/interest, it is preferable that you do so.

The author's relationships/activities/interests should be defined broadly. For example, if your manuscript pertains to the epidemiology of hypertension, you should declare all relationships with manufacturers of antihypertensive medication, even if that medication is not mentioned in the manuscript.

In item #1 below, report all support for the work reported in this manuscript without time limit. For all other items, the time frame for disclosure is the past 36 months.

		**Name all entities with whom you have this relationship or indicate none (add rows as needed)**	**Specifications/Comments (e.g., if payments were made to you or to your institution)**
**Time frame: Since the initial planning of the work**			
1	All support for the present manuscript (e.g., funding, provision of study materials, medical writing, article processing charges, etc.) **No time limit for this item.**	☒ None	
			Click the tab key to add additional rows.
**Time frame: past 36 months**			
**2**	Grants or contracts from any entity (if not indicated in item #1 above).	☐ **None**	
		Associate Editor, Journal of Crohn's and Colitis	Associate editor stipend paid to me
**3**	Royalties or licenses	☐ **None**	
		Principal investigator in development of Pediatric Ulcerative Colitis Activity Index (PUCAI) (Copyright held by Sickkids Hospital Research Institute)	Royalties for use in industry-sponsored studies shared with Sickkids Hospital Research Institute, Dr. Dan Turner, and Dr. Anthony Otley
		Co-principal investigator for development of TUMMY-UC (patient-reported outcome measure)	Royalties for use in industry-sponsored studies shared with Shaare Zedek Hospital, Dr. Dan Turner, Dr. Gili Focht

		Name all entities with whom you have this relationship or indicate none (add rows as needed)	Specifications/Comments (e.g., if payments were made to you or to your institution)
4	Consulting fees	☐ None	
		Lilly	Consulting fees paid to me (steering committee for mirikizumab paediatric clinical trials
		Janssen	Advisory board member (DEVELOP safety registry) and golimumab steering committee member; consulting fees paid to me
		Abbvie	Advisory board member (CAPE safety registry); consulting fees paid to me
5	Payment or honoraria for lectures, presentations, speakers bureaus, manuscript writing or educational events	☐ None	
		Takeda	Speaker honoraria
		Abbvie	Speaker honoraria
		Janssen	Speaker honoraria
6	Payment for expert testimony	☒ None	
7	Support for attending meetings and/or travel	☒ None	
8	Patents planned, issued or pending	☒ None	
9	Participation on a Data Safety Monitoring Board or Advisory Board	☐ None	
		Janssen DEVELOP safety registry advisory board (infliximab)	As above

		Name all entities with whom you have this relationship or indicate none (add rows as needed)	Specifications/Comments (e.g., if payments were made to you or to your institution)
		Abbvie CAPE safety registry advisory board (adalimumab)	As above
		Data safety monitoring Marvel and mini-marvel trials (university of Edinburgh)	
**10**	Leadership or fiduciary role in other board, society, committee or advocacy group, paid or unpaid	☒ **None**	
**11**	Stock or stock options	☒ **None**	
**12**	Receipt of equipment, materials, drugs, medical writing, gifts or other services	☒ **None**	
**13**	Other financial or non-financial interests	☐ **None**	

Please place an "X" next to the following statement to indicate your agreement:

☒ I certify that I have answered every question and have not altered the wording of any of the questions on this form.

ICMJE DISCLOSURE FORM

**Date:**	9/4/2024
**Your Name:**	Eytan Wine
**Manuscript Title:**	Response from the Canadian Children IBD Network to the US FDA Draft Guidance for Industry on Pediatric Inflammatory Bowel Disease: Developing Drugs for Treatment
**Manuscript Number (if known):**	JCAG-2024-0054

In the interest of transparency, we ask you to disclose all relationships/activities/interests listed below that are related to the content of your manuscript. "Related" means any relation with for-profit or not-for-profit third parties whose interests may be affected by the content of the manuscript. Disclosure represents a commitment to transparency and does not necessarily indicate a bias. If you are in doubt about whether to list a relationship/activity/interest, it is preferable that you do so.

The author's relationships/activities/interests should be defined broadly. For example, if your manuscript pertains to the epidemiology of hypertension, you should declare all relationships with manufacturers of antihypertensive medication, even if that medication is not mentioned in the manuscript.

In item #1 below, report all support for the work reported in this manuscript without time limit. For all other items, the time frame for disclosure is the past 36 months.

		**Name all entities with whom you have this relationship or indicate none (add rows as needed)**	**Specifications/Comments (e.g., if payments were made to you or to your institution)**
**Time frame: Since the initial planning of the work**			
1	All support for the present manuscript (e.g., funding, provision of study materials, medical writing, article processing charges, etc.) **No time limit for this item.**	☒ None	
		N/A	
			Click the tab key to add additional rows.
**Time frame: past 36 months**			
**2**	Grants or contracts from any entity (if not indicated in item #1 above).	☐ **None**	
		CIHR	
		Weston Foundation	
		IMAGINE SPOR Network	
**3**	Royalties or licenses	☐ **None**	
		N/A	

		Name all entities with whom you have this relationship or indicate none (add rows as needed)	Specifications/Comments (e.g., if payments were made to you or to your institution)
**4**	Consulting fees	☐ **None**	
		Nestle Health Sciences	Payments made to me
		BioJamp	Payments made to me
		Pfizer	Payments made to me
		AbbVie	Payments made to me
**5**	Payment or honoraria for lectures, presentations, speakers bureaus, manuscript writing or educational events	☐ **None**	
		Nestle Health Sciences	Payments made to me
		Janssen	Payments made to me
		Mead Johnson Nutrition	Payments made to me
		AbbVie	Payments made to me
**6**	Payment for expert testimony	☒ **None**	
**7**	Support for attending meetings and/or travel	☒ **None**	
**8**	Patents planned, issued or pending	☒ **None**	
**9**	Participation on a Data Safety Monitoring Board or Advisory Board	☒ **None**	
**10**	Leadership or fiduciary role in other board, society, committee or advocacy group, paid or unpaid	☒ **None**	

		Name all entities with whom you have this relationship or indicate none (add rows as needed)	Specifications/Comments (e.g., if payments were made to you or to your institution)
**11**	Stock or stock options	☒ **None**	
**12**	Receipt of equipment, materials, drugs, medical writing, gifts or other services	☒ **None**	
**13**	Other financial or non-financial interests	☒ **None**	

Please place an "X" next to the following statement to indicate your agreement:

☒ I certify that I have answered every question and have not altered the wording of any of the questions on this form.

ICMJE DISCLOSURE FORM

**Date:**	9/6/2024
**Your Name:**	Jennifer deBruyn
**Manuscript Title:**	Response from the Canadian Children IBD Network to the US FDA Draft Guidance for Industry on Pediatric Inflammatory Bowel Disease: Developing Drugs for Treatment
**Manuscript Number (if known):**	JCAG-2024-0054

In the interest of transparency, we ask you to disclose all relationships/activities/interests listed below that are related to the content of your manuscript. "Related" means any relation with for-profit or not-for-profit third parties whose interests may be affected by the content of the manuscript. Disclosure represents a commitment to transparency and does not necessarily indicate a bias. If you are in doubt about whether to list a relationship/activity/interest, it is preferable that you do so.

The author's relationships/activities/interests should be defined broadly. For example, if your manuscript pertains to the epidemiology of hypertension, you should declare all relationships with manufacturers of antihypertensive medication, even if that medication is not mentioned in the manuscript.

In item #1 below, report all support for the work reported in this manuscript without time limit. For all other items, the time frame for disclosure is the past 36 months.

		**Name all entities with whom you have this relationship or indicate none (add rows as needed)**	**Specifications/Comments (e.g., if payments were made to you or to your institution)**
**Time frame: Since the initial planning of the work**			
1	All support for the present manuscript (e.g., funding, provision of study materials, medical writing, article processing charges, etc.) **No time limit for this item.**	☒ None	
			Click the tab key to add additional rows.
**Time frame: past 36 months**			
**2**	Grants or contracts from any entity (if not indicated in item #1 above).	☒ **None**	
**3**	Royalties or licenses	☒ **None**	

		Name all entities with whom you have this relationship or indicate none (add rows as needed)	Specifications/Comments (e.g., if payments were made to you or to your institution)
**4**	Consulting fees	☒ **None**	
**5**	Payment or honoraria for lectures, presentations, speakers bureaus, manuscript writing or educational events	☒ **None**	
**6**	Payment for expert testimony	☒ **None**	
**7**	Support for attending meetings and/or travel	☐ **None**	
		Janssen	Janssen paid directly for conference fee and travel
**8**	Patents planned, issued or pending	☒ **None**	
**9**	Participation on a Data Safety Monitoring Board or Advisory Board	☐ **None**	
		Celltrion	Payment made to me
		Abbvie	Payment made to me
**10**	Leadership or fiduciary role in other board, society, committee or advocacy group, paid or unpaid	☐ **None**	
		Canadian Children IBD Network	Unpaid

		Name all entities with whom you have this relationship or indicate none (add rows as needed)	Specifications/Comments (e.g., if payments were made to you or to your institution)
**11**	Stock or stock options	☒ **None**	
**12**	Receipt of equipment, materials, drugs, medical writing, gifts or other services	☒ **None**	
**13**	Other financial or non-financial interests	☒ **None**	

Please place an "X" next to the following statement to indicate your agreement:

☒ I certify that I have answered every question and have not altered the wording of any of the questions on this form.

ICMJE DISCLOSURE FORM

Date: 9/6/2024

Your Name: Eileen Crowley

Manuscript Title: Response from the Canadian Children IBD Network to the US FDA Draft Guidance for Industry on Pediatric Inflammatory Bowel Disease: Developing Drugs for Treatment

Manuscript Number (if known): Click or tap here to enter text.

In the interest of transparency, we ask you to disclose all relationships/activities/interests listed below that are related to the content of your manuscript. "Related" means any relation with for-profit or not-for-profit third parties whose interests may be affected by the content of the manuscript. Disclosure represents a commitment to transparency and does not necessarily indicate a bias. If you are in doubt about whether to list a relationship/activity/interest, it is preferable that you do so.

The author's relationships/activities/interests should be defined broadly. For example, if your manuscript pertains to the epidemiology of hypertension, you should declare all relationships with manufacturers of antihypertensive medication, even if that medication is not mentioned in the manuscript.

In item #1 below, report all support for the work reported in this manuscript without time limit. For all other items, the time frame for disclosure is the past 36 months.

		**Name all entities with whom you have this relationship or indicate none (add rows as needed)**	**Specifications/Comments (e.g., if payments were made to you or to your institution)**
**Time frame: Since the initial planning of the work**			
1	All support for the present manuscript (e.g., funding, provision of study materials, medical writing, article processing charges, etc.) **No time limit for this item.**	☒ None	
			Click the tab key to add additional rows.
**Time frame: past 36 months**			
**2**	Grants or contracts from any entity (if not indicated in item #1 above).	☐ **None**	
		Significance of magnetic resonance enterography detected sacroiliitis in pediatric inflammatory bowel disease: A retrospective cohort study.	Internal Research Grant Fund Competition, Children's Health Research Institute (CHRI), London, Ontario.
		Improving access to Transition Support. Children's Health Foundation, LHSC	Children's Health Foundation, LHSC
		Musculoskeletal Manifestations of IBD	Crohn's and Colitis Canada, Grants in Aid of Research
		Shared Decision Making in Paediatric Inflammatory Bowel Disease	AMOSO Innovation Fund
		Reliability of MRI Indices for Evaluating Perianal Fistulizing Crohn's Disease Activity in a Paediatric Cohort	FLIBD Fellowship Grant Project

		Name all entities with whom you have this relationship or indicate none (add rows as needed)	Specifications/Comments (e.g., if payments were made to you or to your institution)
		Monogenic manifestations of IBD	Canadian Institutes of Health Research (CIHR) grant funding, SickKids
3	Royalties or licenses	☒ **None**	
4	Consulting fees	☐ **None**	
		Alimentiv Inc	Consulting (payments to me)
		Sanofi	Consulting (payments to me)
		Pfizer	Consulting (payments to me)
		Abbvie	Consulting (payments to me)
5	Payment or honoraria for lectures, presentations, speakers bureaus, manuscript writing or educational events	☐ **None**	
		Pfizer	Stipend for lecture at educational event (payment to me)
		Abbvie	Stipend for lecture at educational event (payment to me)
6	Payment for expert testimony	☒ **None**	
7	Support for attending meetings and/or travel	☐ **None**	
		Pfizer	ECCO conference 2023
8	Patents planned, issued or pending	☒ **None**	
9	Participation on a Data Safety	☒ **None**	

		Name all entities with whom you have this relationship or indicate none (add rows as needed)	Specifications/Comments (e.g., if payments were made to you or to your institution)
	Monitoring Board or Advisory Board		
**10**	Leadership or fiduciary role in other board, society, committee or advocacy group, paid or unpaid	☒ **None**	
**11**	Stock or stock options	☒ **None**	
**12**	Receipt of equipment, materials, drugs, medical writing, gifts or other services	☐ **None**	
		Pfizer	Support for intestinal ultrasound machine – loan to hospital.
**13**	Other financial or non-financial interests	☒ **None**	

Please place an "X" next to the following statement to indicate your agreement:

☒ I certify that I have answered every question and have not altered the wording of any of the questions on this form.
